# Spatiotemporal distribution of newly diagnosed echinococcosis patients in Sichuan Province, China, 2013–2024

**DOI:** 10.1371/journal.pntd.0013358

**Published:** 2025-08-04

**Authors:** Wei He, Yang Liu, Bo Zhong, Wenjie Yu, Sha Liao, Guangjia Zhang, Qi Wang, Ruirui Li, Liu Yang, Renxin Yao, Zhongshuang Zhang, Yan Huang, Liying Wang, Qian Wang

**Affiliations:** 1 Institute of Parasitic Diseases Prevention and Control, Sichuan Center for Disease Control and Prevention, Chengdu, China; 2 National Institute of Parasitic Diseases, Chinese Center for Disease Control and Prevention (Chinese Center for Tropical Diseases Research); NHC Key Laboratory of Parasite and Vector Biology, Shanghai, China; NEHU: North Eastern Hill University, INDIA

## Abstract

**Background:**

Echinococcosis is a zoonotic parasitic disease that significantly endangers public health and hinders socioeconomic development. Sichuan Province is a mixed endemic area of cystic echinococcosis (CE) and alveolar echinococcosis (AE), and it has one of the most severe epidemics of echinococcosis in China. In 2012, the national epidemiological sampling survey of echinococcosis revealed that the prevalence rate of echinococcosis among the population in Sichuan Province was 1.08%, significantly higher than the national average (0.24%). In this study, the spatiotemporal distribution characteristics of newly diagnosed patients with echinococcosis in Sichuan Province from 2013 to 2024 were analyzed, providing a reference for identifying key areas of echinococcosis and developing targeted prevention and control strategies.

**Methods:**

Data on the number of individuals screened for echinococcosis and the number of newly diagnosed patients in endemic counties were obtained from the annual reports of the Sichuan Provincial Echinococcosis Control Program between 2013 and 2024. The detection rate of newly diagnosed echinococcosis patients was calculated for each year. County-level electronic maps of Sichuan Province were downloaded from the National Geographic Information Public Service Platform. The spatial distribution map of the detection rate of newly diagnosed patients with echinococcosis in Sichuan Province was created using ArcGIS software, and global and local spatial autocorrelation analyses were conducted. Spatiotemporal scanning analysis of the detection rate of newly diagnosed echinococcosis patients in Sichuan Province was conducted using SaTScan software.

**Results:**

From 2013 to 2024, the total detection rate of newly diagnosed patients with echinococcosis in the endemic areas of Sichuan Province was 55.77/100,000, with the detection rates of CE and AE being 36.71/100,000 and 19.88/100,000, respectively. The total detection rate of newly diagnosed patients with echinococcosis, as well as that of CE and AE, demonstrated a decreasing trend year by year (*χ*^*2 *^= 1,054.785, 925.936, 196.018; **P* *< 0.001). The detection rate of CE was higher than that of AE between 2013 and 2021; however, this trend was reversed between 2022 and 2024. In terms of spatial distribution, areas with higher detection rates were primarily concentrated in the northwestern part of Sichuan Province, whereas areas with lower detection rates were mainly distributed in the southeastern part of the province. The global spatial autocorrelation analysis results revealed that the total detection rate of newly diagnosed echinococcosis patients exhibited spatial clustering in 2013–2016, 2018, 2020, and 2024. The detection rate of newly diagnosed CE patients exhibited spatial clustering in 2013, 2014, and 2022. The detection rate of newly diagnosed patients with AE exhibited spatial clustering in 2013–2018, 2020, and 2023. Local spatial autocorrelation analyses revealed that the “high-high” clustering areas of the total detection rates, CE and AE detection rates were concentrated in the northwestern and northern parts of the endemic areas, while the “low-low” clustering areas were concentrated in the southeastern parts of the endemic areas. Spatiotemporal scanning analysis revealed that the most important clusters of newly total diagnosed echinococcosis and CE patients in Sichuan Province were mainly located in Shiqu, Seda, Baiyu, Ganzi, Dege, Xinlong, Luhuo, Aba, and Rangtang counties. The most important clusters of patients with AE were mainly in Shiqu, Seda, Baiyu, Ganzi, Dege, and Rangtang counties.

**Conclusion:**

The results demonstrated that the detection rate of newly diagnosed patients with echinococcosis in Sichuan Province decreased annually between 2013 and 2024, exhibiting significant spatial clustering. The western Sichuan Qinghai Tibet Plateau region is a “hot spot” for echinococcosis in the Sichuan Province population. It is recommended that relevant departments develop precise prevention and control strategies for the current areas of clustering.

## Author summary

Echinococcosis is a zoonotic parasitic disease that significantly endangers public health and hinders socioeconomic development. Sichuan Province is a mixed endemic area of cystic echinococcosis (CE) and alveolar echinococcosis (AE), and it has one of the most severe epidemics of echinococcosis in China. Identifying spatiotemporal hotspots is critical for targeted interventions. We analyzed 12 years (2013–2024) of surveillance data from Sichuan’s Echinococcosis Control Program, covering 35 endemic counties and 3,215 newly diagnosed patients. Spatial autocorrelation (global/local Moran’s *I*) and spatiotemporal scan statistics (SaTScan) were applied to detect clustering patterns. The overall detection rate declined significantly, but CE predominated until 2021, after which AE surpassed it. Persistent “high-high” clusters were concentrated in northwestern counties, while southeastern areas showed “low-low” clustering. Significant spatiotemporal aggregation occurred during 2013–2016 for total/CE cases and extended to 2018 for AE. Key drivers included ecological factors, limited healthcare access, and socioeconomic practices. The Sichuan-Tibet Plateau remains a high-risk epicenter requiring prioritized control. We recommend precision strategies: dog management and rodent control in AE-endemic clusters, AI-enhanced diagnostics, dynamic spatial warning systems, and differentiated interventions for high/low-risk zones. These approaches align with Healthy China 2030 goals.

## Introduction

Echinococcosis, commonly known as hydatid disease, is a zoonotic parasitic disease caused by the larvae of the *Echinococcus* tapeworm, which parasitizes humans or animals [1]. In China, there are two types of echinococcosis: cystic echinococcosis (CE) caused by *Echinococcus granulosus* larvaeand alveolar echinococcosis (AE) caused by *Echinococcus multilocularis* larvae [2]. Echinococcosis can damage several vital organs of the human body, including the liver, lungs, brain, kidneys, and bones. If the cyst ruptures, it can cause anaphylactic shock, which can be life-threatening in severe cases. The epidemiology of echinococcosis is complex, involving various host species. Among them, CE not only poses a threat to human beings but also seriously jeopardizes the health of livestock in endemic areas, thereby affecting the development of the livestock industry. The mortality rate of AE is high, with a 10-year fatality rate of up to 94% if untreated; therefore, it is known as “worm cancer” [3].

Echinococcosis endemic areas in China are mainly distributed in the western and Tibetan Plateau regions, with Sichuan Province being one of the most severely affected regions [4]. In 2012, the national epidemiological survey on echinococcosis confirmed that echinococcosis in Sichuan Province was prevalent in 35 counties across four cities (prefectures): Ganzi, Aba, Ya’an, and Liangshan. The population prevalence rate of echinococcosis was 1.08%. Among them, the prevalence rate was higher than 1% in 10 counties, and the mixed epidemics of the two types of echinococcosis were observed in 15 counties. In these counties, the prevalence of CE was 0.81%, and that of AE was 0.28%. Overall, the disease exhibited higher prevalence rates among females, older age groups, Tibetans, religious people, farmers, herders, and people with low literacy levels [2]. Echinococcosis has a long incubation period and a prolonged course and is significantly harmful. Currently, no specific drug is available, resulting in great pain and a heavy economic burden for patients and their families, as well as a significant loss of livestock production. It is one of the main causes of poverty and poverty recurrence among residents of endemic areas in Sichuan Province, limiting the local social and economic development. Previous cross-sectional surveys have revealed the spatial distribution characteristics of echinococcosis prevalence in Sichuan Province, characterized by persistent hotspots in the northwestern plateau and scattered cases in southeastern agricultural areas, correlating with agro-pastoral transitions and altitudinal gradients [5]. However, the risk areas for sustained transmission of echinococcosis have not been accurately identified in both spatial and temporal dimensions. Additionally, the detection rate of newly diagnosed patients has not been used to identify high-risk areas. As a result, it is imperative to comprehensively analyze the distribution characteristics of echinococcosis in Sichuan Province, especially the spatial and temporal distribution of newly diagnosed patients, to develop precise prevention and control strategies and identify key areas for prevention and control.

The northwestern high-altitude plateau of Sichuan is a high-cold climate zone, characterized by significant elevation differences and unique vertical climate variations. It features a cold climate, with dry and warm valleys and cold, humid mountainous regions. Winters are cold, summers are cool, and the region experiences insufficient heat and moisture. The annual average temperature ranges from 4 to 12 °C, with an annual precipitation of 500–900 mm. The weather is mostly clear with abundant sunshine, with an average of 1,600–2,600 h of sunlight annually [6]. The vegetation in most areas of the northwestern high-altitude plateau consists mainly of grasslands dominated by grasses and sedges, with alpine and subalpine meadows as the primary types [7]. Over 190 species of ungulates, rodents, and carnivorous mammals inhabit the region, making it a rich reservoir of animal hosts for *Echinococcus* transmission [8].

This study aimed to analyze the spatial and temporal distribution of newly diagnosed patients in Sichuan Province between 2013 and 2024, identify distribution patterns, detect high-risk areas, and provide a scientific basis for formulating precise and effective prevention and control strategies for echinococcosis.

## Methods

### Ethics statement

This study included population-based echinococcosis screening data from all endemic counties in Sichuan Province between 2013 and 2024. The screening activities were approved by the Ethics Committee of the Sichuan Provincial Center for Disease Control and Prevention (approval no. SCCDCIRB: 2024-018). The participants were provided with detailed explanations of the screening content, objectives, potential consequences, and benefits. Informed consent was obtained orally from the patients after explaining the relevant content and risks. Furthermore, the personal information and privacy of the patients were strictly protected.

### Survey areas

This study included all 35 echinococcosis-endemic counties in Sichuan Province, including 18 in Ganzi prefecture, 13 in Aba prefecture, Muli Tibetan autonomous county, Yuexi county in Liangshan prefecture, as well as Tianquan and Baoxing counties in Ya’an city.

### Data source

Data on the number of individuals screened for echinococcosis, newly diagnosed echinococcosis cases, and lesion classification results from 2013 to 2024 were obtained from the annual reports of the Sichuan Provincial Echinococcosis Control Program (managed by Sichuan Provincial for Disease Control and Prevention). Population-based ultrasound screening for human echinococcosis is conducted annually in the survey areas. Comprehensive population screenings are performed every 3–5 years to ensure complete case ascertainment. Quality control included standardized ultrasonographer training and monthly expert review of cases. The detection rate of newly diagnosed echinococcosis cases was calculated using the following formula:

(Number of newly detected echinococcosis cases/number of people screened) × 100,000. Similarly, the detection rates for newly diagnosed CE and AE cases were calculated using the same formula.

Newly identified echinococcosis cases in this study were operationally defined as patients who received their initial diagnosis and were officially reported within the current calendar year, and had documented negative screening results throughout the preceding three-year surveillance period. This case definition ensures that the annual tally of incident cases reliably approximates the true incidence of recent infections within the study population. All newly diagnosed echinococcosis cases were confirmed according to the Diagnostic Criteria for Hydatid Disease (WS 257-2006) [9]. A county-level database was established for newly diagnosed echinococcosis cases and detection rates. All cases were logically verified before inclusion in the statistical analysis.

The county-level electronic map of Sichuan Province was downloaded from the National Geographic Information Public Service Platform, with geographic data based on the 1:1,000,000 vector map of Sichuan Province.

### Data analysis and statistics

#### Spatial autocorrelation analysis.

To evaluate the presence of spatial autocorrelation across the entire study area for the specified attributes, global spatial autocorrelation analysis was performed using Global Moran’s *I* index. The formula for Global Moran’s *I* is:


$$I=\frac{n\sum_{i=1}^{n}\sum_{j=1}^{n}w_{ij}\left(x_{i}-\bar{x}\right)\left(x_{j}-\bar{x}\right)}{\sum_{i=1}^{n}\sum_{j=1}^{n}w_{ij}\sum_{i=1}^{n}{\left(x_{i}-\vec{x}\right)}^{2}}$$

where:

*n* refers to the total number of observed values.
$x_{i}$ and $x_{j}$ represent the attribute values at locations *i* and *j,* i≠j.
$\overset{―}{x}$ refers to the average of observed values at all n positions, $\overset{―}{x}=\frac{1}{n}\sum_{i=1}^{n}x_{i}$*.*
$w_{ij}$ refers to the element value of symmetric binomial distribution spatial weight matrix, which measures the influence and relationship between spatial positions *i* and *j* [10,11].

Global Moran’s *I* values range between -1.0 and 1.0. A positive index signifies positive spatial autocorrelation (clustering of similar values), with magnitude indicating its strength. Conversely, a negative index indicates negative spatial autocorrelation (dispersion or dissimilarity between neighbors), where lower values reflect greater spatial heterogeneity. An index value near zero suggests a spatially random pattern. This analysis employed a queen contiguity spatial weights matrix to define adjacency between counties [12,13].

Global spatial autocorrelation analysis was conducted first. If global spatial autocorrelation is present, a local spatial autocorrelation analysis is performed. Local spatial autocorrelation analysis is a statistical method used to detect spatial autocorrelation in localized regions, thereby identifying spatial differences caused by spatial correlations. This helps determining spatial hotspots or high-incidence areas of attribute values, thereby complementing the limitations of global spatial autocorrelation analysis.

To identify localized clustering patterns in the observed variables, we employed the Local Moran’s *I* statistic. The formula is expressed as follows:


$$\begin{array}{c} I_{i}=\frac{y_{i}-\overset{―}{y}}{\delta^{2}}\sum {\mathrm{\backslash nolimits}}_{j=1}^{n}W_{ij}\left(y_{j}-\overset{―}{y}\right) \end{array}$$

In the formula, $\delta^{2}=\frac{1}{n}\sum_{i=1}^{n}{\left(y_{i}-\overset{―}{y}\right)}^{2}$*,*$\overset{―}{\;y}=\frac{1}{n}\sum_{i=1}^{n}y_{i}$

Within this study, local spatial autocorrelation analysis was performed for years with significant global spatial autocorrelation to identify localized clusters of newly diagnosed echinococcosis cases. Local Indicators of Spatial Association (LISA) cluster maps were generated to illustrate four types of local correlations: “high-low outliers”, “low-high outliers”, “high-high clusters”, and “low-low clusters”. “High-high clusters” refer to areas with high detection rates of newly diagnosed echinococcosis cases, which are prioritized for detection and intervention. “Low-low clusters” refer to areas with low detection rates, while “high-low” and “low-high” outliers indicate significant differences in detection rate between the target area and its neighboring regions [14].

#### Spatiotemporal scan analysis.

Spatiotemporal scan analysis, also known as spatiotemporal scan statistics (STSS). involves scanning the entire study area and time period using a dynamically moving cylindrical window (where the base represents the geographic area and the height represents the time duration). For each window, it calculates the ratio of the observed number of events to the expected number (typically based on background population risk) and identifies space-time clusters where the observed count is significantly higher than expected using a Likelihood Ratio Test. It has been widely used to detect spatiotemporal clusters of diseases. Unlike spatial clusters, spatiotemporal clusters elucidate the spatial extent and temporal duration of clustering. In this study, spatiotemporal scan analysis was used to identify spatiotemporal clusters of newly diagnosed echinococcosis cases, the centers, and the radii of the clustered regions, and to determine whether these clusters were due to random variation. Assuming that the detection rate of newly diagnosed echinococcosis cases followed a Poisson distribution, a discrete Poisson model was used for the spatiotemporal scan analysis. A scanning window radius covering 50% of the population at risk was selected, with the window moving in annual time intervals. The maximum scanning time was set to 50% of the study period, and Monte Carlo simulations were performed 999 times to calculate the relative risk (RR), logarithmic likelihood ratio (LLR), and *P*-values. When **P* *< 0.05, a higher LLR value indicated a greater likelihood of spatiotemporal clustering within the window [15].

### Statistical methods

In this study, WPS Office 2023 software was used for statistical analysis and linear trend *χ*^*2*^ tests of the detection rates of newly diagnosed echinococcosis cases in Sichuan Province between 2013 and 2024. A **P* *< 0.05 was considered statistically significant. ArcGIS version 10.3 (ESRI, Redlands, California, USA) was used for spatial distribution mapping, global spatial autocorrelation, and local spatial autocorrelation analyses. SaTScan 10.1.2 software (Management Information Services, Maryland, USA) was used for the STSS, and the results were visualized using ArcGIS 10.3.

## Results

### Basic information

#### Temporal characteristics.

**Fig 1:**
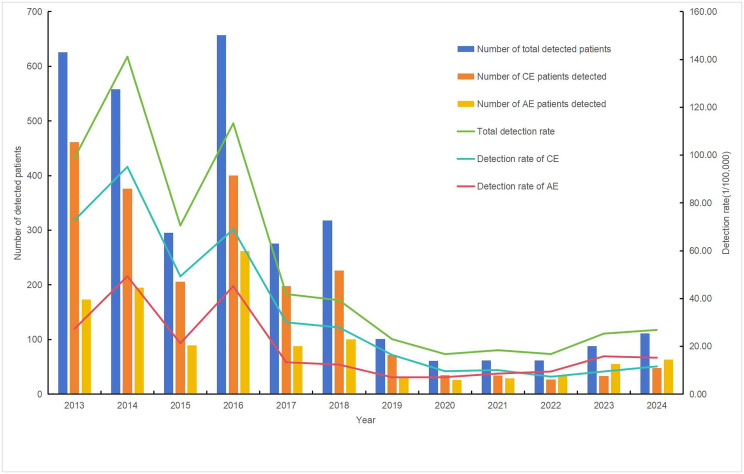
Number and detection rate of newly detected echinococcosis patients in Sichuan Province (2013–2024).

From 2013 to 2024, 5,764,382 individuals were screened for echinococcosis in endemic areas of Sichuan Province. A total of 3,215 new echinococcosis cases were diagnosed, comprising 2,116 cases of CE, 1,146 cases of AE, and 47 cases exhibiting both forms (classified as mixed echinococcosis). The detection rates of CE and AE were 36.71 and 19.88 per 100,000, respectively. The annual detection rates of newly diagnosed echinococcosis cases (including both cystic and alveolar types) demonstrated a declining trend over the years (*χ*^*2*^ = 1,054.785, **P* *< 0.001). From 2013 to 2021, the detection rate of CE was higher than that of AE. However, the detection rate of AE surpassed that of CE between 2022 and 2024 (Table 1; Fig 1).

**Table 1:** Detection status of newly diagnosed echinococcosis patients in Sichuan Province between 2013 and 2024.

Year	Number of screened	Number of total detected patients	Total detection rate (1/100,000)	CE		AE	
				Number of detected patients	Detection rate (1/100,000)	Number of detected patients	Detection rate (1/100,000)
2013	633567	626	98.81	461	72.76	173	27.31
2014	395327	558	141.15	376	95.11	195	49.33
2015	417910	295	70.59	206	49.29	89	21.30
2016	579882	657	113.30	400	68.98	262	45.18
2017	659267	276	41.86	198	30.03	88	13.35
2018	809376	318	39.29	226	27.92	100	12.36
2019	438572	101	23.03	72	16.42	31	7.07
2020	364003	61	16.76	35	9.62	26	7.14
2021	337058	62	18.39	34	10.09	29	8.60
2022	369193	62	16.79	27	7.31	35	9.48
2023	347256	88	25.34	33	9.50	55	15.84
2024	412971	111	26.88	48	11.62	63	15.26

CE, Cystic echinococcosis; AE, Alveolar echinococcosis.

#### Spatial distribution characteristics.

**Fig 2:**
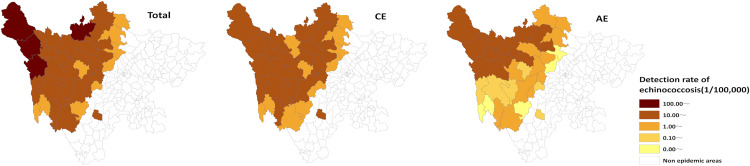
Regional distribution of annual average detection rates of echinococcosis in Sichuan Province (2013–2024). The base layer is from https://www.webmap.cn/mapDataAction.do?method=forw&resType=5&storeId=2&storeName=%E5%9B%BD%E5%AE%B6%E5%9F%BA%E7%A1%80%E5%9C%B0%E7%90%86%E4%BF%A1%E6%81%AF%E4%B8%AD%E5%BF%83&fileId=BA420C422A254198BAA5ABAB9CAAFBC1 with credit to National Catalogue Service For Geographic Information.

**Fig 3:**
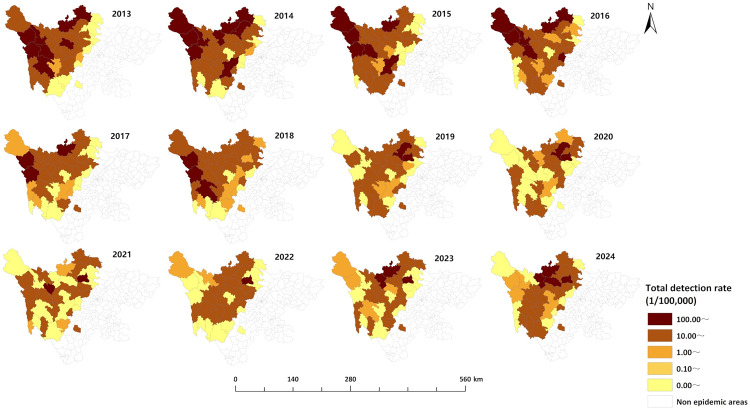
Regional distribution of overall detection rate of echinococcosis in Sichuan Province (2013–2024). The base layer is from https://www.webmap.cn/mapDataAction.do?method=forw&resType=5&storeId=2&storeName=%E5%9B%BD%E5%AE%B6%E5%9F%BA%E7%A1%80%E5%9C%B0%E7%90%86%E4%BF%A1%E6%81%AF%E4%B8%AD%E5%BF%83&fileId=BA420C422A254198BAA5ABAB9CAAFBC1 with credit to National Catalogue Service For Geographic Information.

**Fig 4:**
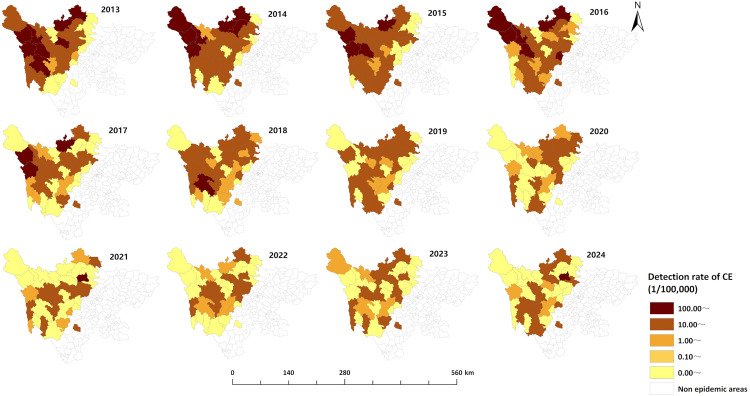
Regional distribution of annual detection rates of CE in Sichuan Province (2013–2024). The base layer is from https://www.webmap.cn/mapDataAction.do?method=forw&resType=5&storeId=2&storeName=%E5%9B%BD%E5%AE%B6%E5%9F%BA%E7%A1%80%E5%9C%B0%E7%90%86%E4%BF%A1%E6%81%AF%E4%B8%AD%E5%BF%83&fileId=BA420C422A254198BAA5ABAB9CAAFBC1 with credit to National Catalogue Service For Geographic Information.

**Fig 5:**
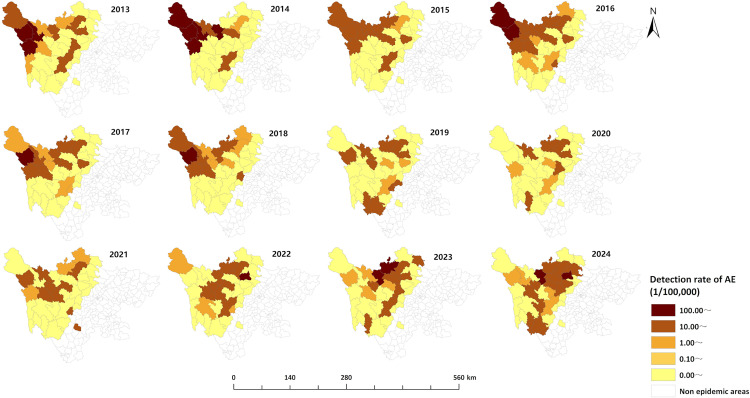
Regional distribution of annual detection rates of AE in Sichuan Province (2013–2024). The base layer is from https://www.webmap.cn/mapDataAction.do?method=forw&resType=5&storeId=2&storeName=%E5%9B%BD%E5%AE%B6%E5%9F%BA%E7%A1%80%E5%9C%B0%E7%90%86%E4%BF%A1%E6%81%AF%E4%B8%AD%E5%BF%83&fileId=BA420C422A254198BAA5ABAB9CAAFBC1 with credit to National Catalogue Service For Geographic Information.

From 2013 to 2024, newly diagnosed echinococcosis cases were detected in all 35 endemic counties of Sichuan Province. The top five counties (districts) with the highest detection rates were Baiyu (157.75 per 100,000), Shiqu (148.28 per 100,000), Dege (143.33 per 100,000), Aba (103.05 per 100,000), and Xinlong (98.93 per 100,000). The top five counties (districts) with the highest average annual detection rates of CE were Baiyu (92.79 per 100,000), Xinlong (88.55 per 100,000), Dege (83.61 per 100,000), Shiqu (77.10 per 100,000), and Aba (75.64 per 100,000). The top five counties (districts) with the highest average annual detection rates for AE were Rangtang (77.03 per 100,000), Shiqu (73.90 per 100,000), Baiyu (67.74 per 100,000), Dege (62.79 per 100,000), and Ganzi (46.70 per 100,000). The areas with higher detection rates were mainly concentrated in the northwest of Sichuan Province, which is the endemic area for echinococcosis, while the areas with lower detection rates were mostly located in the southeastern part of the endemic area (Fig 2). The detection rates of newly diagnosed echinococcosis cases in most areas declined over time. However, it is noteworthy that Rangtang, Heishui, and Aba counties in the Aba prefecture demonstrated an increasing trend in recent years (Figs 3–5). The cases of mixed echinococcosis were mainly distributed in Shiqu, Baiyu, Ganzi, Dege, Xinlong, Aba and Jinchuan counties.

### Spatial autocorrelation analysis

#### Global spatial autocorrelation analysis.

The global spatial autocorrelation analysis revealed that the overall detection rate of newly diagnosed echinococcosis cases exhibited spatial clustering in 2013–2016, 2018, 2020, and 2024 (Moran’s *I* = 0.292, 0.241, 0.150, 0.091, 0.157, 0.227, 0.114; **Z* *> 1.96, **P* *< 0.05). The detection rate of newly diagnosed CE cases exhibited spatial clustering in 2013, 2014, and 2022 (Moran’s *I* = 0.213, 0.221, 0.069; **Z* *> 1.96, **P* *< 0.05). The detection rate of newly diagnosed AE cases exhibited spatial clustering in 2013–2018, 2020, and 2023 (Moran’s *I* = 0.223, 0.219, 0.197, 0.074, 0.122, 0.260, 0.158, 0.126; **Z* *> 1.96, **P* *< 0.05) (Table 2).

**Table 2:** Global spatial autocorrelation analysis of newly diagnosed echinococcosis patients in Sichuan Province between 2013 and 2024.

Year	Moran’s *I* value			Expected index			Variance			Z value			P value		
	Total detection rate	Detection rate of CE	Detection rate of AE	Total detection rate	Detection rate of CE	Detection rate of AE	Total detection rate	Detection rate of CE	Detection rate of AE	Total detection rate	Detection rate of CE	Detection rate of AE	Total detection rate	Detection rate of CE	Detection rate of AE
2013	0.292	0.213	0.224	−0.029	−0.029	−0.029	0.006	0.007	0.005	4.088	2.990	3.547	<0.001	0.003	<0.001
2014	0.241	0.221	0.219	−0.029	−0.029	−0.029	0.006	0.006	0.006	3.531	3.331	3.203	<0.001	<0.001	0.001
2015	0.150	0.109	0.197	−0.029	−0.029	−0.029	0.006	0.006	0.007	2.297	1.811	2.792	0.022	0.070	0.005
2016	0.091	0.059	0.074	−0.029	−0.029	−0.029	0.003	0.006	0.001	2.107	1.152	2.932	0.035	0.249	0.003
2017	0.109	0.066	0.122	−0.029	−0.029	−0.029	0.006	0.006	0.005	1.810	1.235	2.134	0.070	0.217	0.033
2018	0.157	0.096	0.260	−0.029	−0.029	−0.029	0.006	0.006	0.004	2.395	1.566	4.412	0.017	0.117	<0.001
2019	0.062	0.028	0.092	−0.029	−0.029	−0.029	0.007	0.007	0.006	1.124	0.704	1.608	0.261	0.482	0.108
2020	0.227	0.052	0.158	−0.029	−0.029	−0.029	0.006	0.007	0.005	3.247	1.004	2.731	0.001	0.316	0.006
2021	−0.163	−0.087	−0.004	−0.029	−0.029	−0.029	0.007	0.007	0.006	−1.601	−0.693	0.334	0.109	0.489	0.738
2022	0.069	0.135	−0.001	−0.029	−0.029	−0.029	0.004	0.006	0.004	1.493	2.159	0.438	0.135	0.031	0.661
2023	0.090	−0.096	0.126	−0.029	−0.029	−0.029	0.005	0.006	0.004	1.648	−0.843	2.477	0.099	0.399	0.013
2024	0.114	0.046	0.035	−0.029	−0.029	0.029	0.005	0.004	0.004	2.008	1.136	1.072	0.045	0.256	0.284

CE, Cystic echinococcosis; AE, Alveolar echinococcosis.

#### Local spatial autocorrelation analysis.

**Fig 6:**
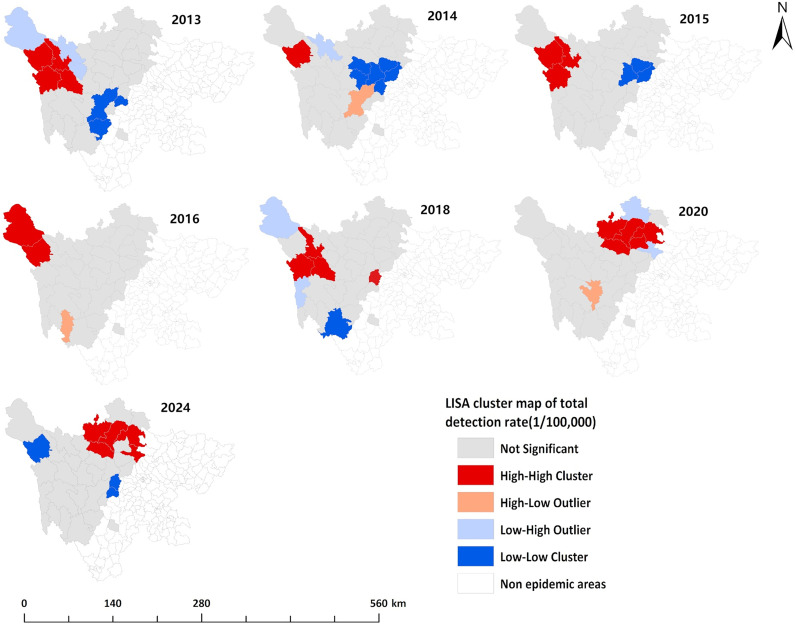
Local spatial autocorrelation analysis of the overall detection rate of echinococcosis in Sichuan Province (2013–2024). The base layer is from https://www.webmap.cn/mapDataAction.do?method=forw&resType=5&storeId=2&storeName=%E5%9B%BD%E5%AE%B6%E5%9F%BA%E7%A1%80%E5%9C%B0%E7%90%86%E4%BF%A1%E6%81%AF%E4%B8%AD%E5%BF%83&fileId=BA420C422A254198BAA5ABAB9CAAFBC1 with credit to National Catalogue Service For Geographic Information.

**Fig 7:**
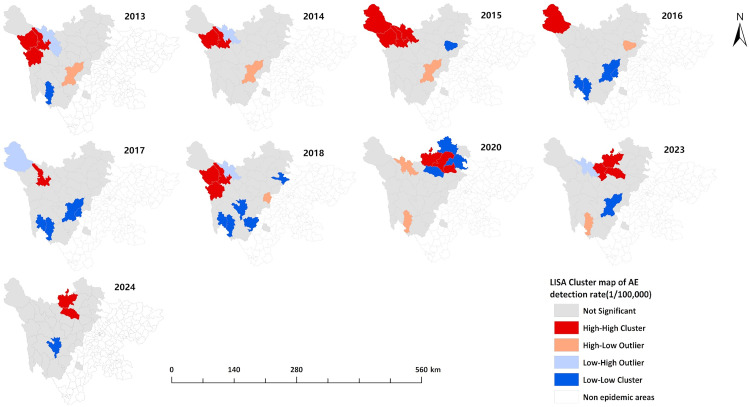
Local spatial autocorrelation analysis of the detection rate of CE in Sichuan Province (2013–2024). The base layer is from https://www.webmap.cn/mapDataAction.do?method=forw&resType=5&storeId=2&storeName=%E5%9B%BD%E5%AE%B6%E5%9F%BA%E7%A1%80%E5%9C%B0%E7%90%86%E4%BF%A1%E6%81%AF%E4%B8%AD%E5%BF%83&fileId=BA420C422A254198BAA5ABAB9CAAFBC1 with credit to National Catalogue Service For Geographic Information.

**Fig 8:**
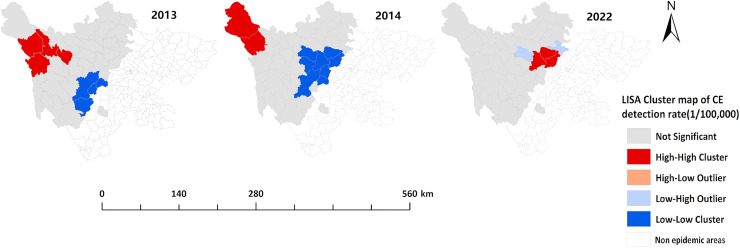
Local spatial autocorrelation analysis of detection rate of AE in Sichuan Province (2013–2024). The base layer is from https://www.webmap.cn/mapDataAction.do?method=forw&resType=5&storeId=2&storeName=%E5%9B%BD%E5%AE%B6%E5%9F%BA%E7%A1%80%E5%9C%B0%E7%90%86%E4%BF%A1%E6%81%AF%E4%B8%AD%E5%BF%83&fileId=BA420C422A254198BAA5ABAB9CAAFBC1 with credit to National Catalogue Service For Geographic Information.

The LISA cluster maps revealed that the number of counties with “high-high” clusters for the overall detection rate of newly diagnosed echinococcosis cases in 2013–2016, 2018, 2020, and 2024 was 4, 1, 3, 2, 4, 5, and 5, respectively. These “high-high” clusters were mainly concentrated in the northwestern and northern parts of the endemic region, including Maerkang, Dege, Ganzi, Baiyu, Xinlong, Shiqu, Baoxing, Aba, Hongyuan, Songpan, Heishui, and Mao counties. For the detection rate of newly diagnosed CE cases, the number of counties with “high-high” clusters in 2013, 2014, and 2022 was 4, 2, and 3, respectively. These clusters were primarily located in the northwestern and northern parts of the endemic region, including Shiqu, Dege, Baiyu, Ganzi, Luhuo, Xiaojin, Li, and Wenchuan counties. For the detection rate of newly diagnosed AE cases, the number of counties with “high-high” clusters in 2013–2018, 2020, and 2023 was 3, 2, 4, 1, 1, 3, 3, and 3, respectively. These clusters were primarily concentrated in the northwestern and northern parts of the endemic region, including Maerkang, Dege, Baiyu, Ganzi, Shiqu, Seda, Aba, Hongyuan, Heishui, and Rangtang counties. The “high-high” clusters gradually shifted from northwest to north over time. The “low-low” clusters were mainly concentrated in the southeastern part of the endemic region (Figs 6–8).

### Spatiotemporal scan analysis

The following results were obtained from the spatiotemporal scan analysis of the overall detection rate of newly diagnosed echinococcosis cases, as well as the detection rates of CE and AE cases from 2013 to 2024.

Overall echinococcosis detection rate: One primary and one secondary cluster were identified. The primary cluster was located in the northwestern part of the endemic region, covering nine counties: Shiqu, Seda, Baiyu, Ganzi, Dege, Xinlong, Luhuo, Aba, and Rangtang. The cluster center was located at 33.21°N, 98.20°E, with a cluster radius of 334.91 km. The cluster period was from 2013–2016 (RR = 7.50, LLR = 1431.55, **P* *< 0.001). The secondary cluster was located in Heishui County, with a cluster period from 2020 to 2024 (RR = 3.58, LLR = 15.99, **P* *< 0.001).

CE detection rate: One primary and one secondary cluster were identified. The primary cluster was located in the northwestern part of the endemic region, covering nine counties: Shiqu, Seda, Baiyu, Ganzi, Dege, Xinlong, Luhuo, Aba, and Rangtang. The cluster center was located at 33.21°N, 98.20°E, with a cluster radius of 334.91 km. The cluster period was from 2013–2016 (RR = 6.46, LLR = 788.92, **P* *< 0.001). The secondary cluster was located in Ruoergai County, with a cluster period from 2013 to 2016 (RR = 3.53, LLR = 48.60, **P* *< 0.001).

**Fig 9:**
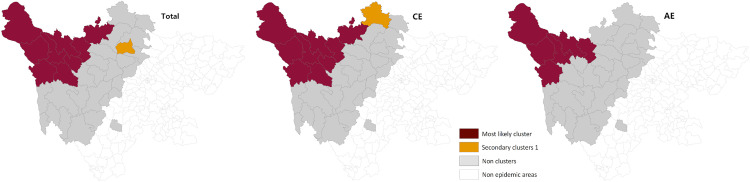
Spatial and temporal aggregation of echinococcosis detection rates in Sichuan Province (2013–2024). The base layer is from https://www.webmap.cn/mapDataAction.do?method=forw&resType=5&storeId=2&storeName=%E5%9B%BD%E5%AE%B6%E5%9F%BA%E7%A1%80%E5%9C%B0%E7%90%86%E4%BF%A1%E6%81%AF%E4%B8%AD%E5%BF%83&fileId=BA420C422A254198BAA5ABAB9CAAFBC1 with credit to National Catalogue Service For Geographic Information.

AE detection rate: One primary cluster was identified, located in the northwestern part of the endemic region, covering six counties: Shiqu, Seda, Baiyu, Ganzi, Dege, and Rangtang. The cluster center was located at 33.21°N, 98.20°E, with a cluster radius of 291.67 km. The cluster period was from 2013–2018 (RR = 13.29, LLR = 881.15, **P* *< 0.001) (Table 3, Fig 9).

**Table 3:** Spatiotemporal scanning analysis results of newly diagnosed echinococcosis patients in Sichuan Province between 2013 and 2024.

Category	Cluster	Cluster areas	Cluster center/radius (km)	Time frame	Number of cases	Expected cases	RR	LLR	P-value
Total	Most likely cluster	9	(33.21 N, 98.20 E)/ 334.91	2013–2016	1647	395	7.50	1431.55	<0.001
	Secondary cluster	1	(32.17 N, 103.05 E)/ 0	2020–2024	29	8	3.58	15.99	<0.001
CE	Most likely cluster	9	(33.21 N, 98.20 E)/ 334.91	2013–2016	1005	260	6.46	788.92	<0.001
	Secondary cluster	1	(33.66 N, 102.88 E)/ 0	2013–2016	91	27	3.53	48.60	<0.001
AE	Most likely cluster	6	33.21 N, 98.20 E)/ 291.67	2013–2018	799	169	13.29	881.15	<0.001

CE, Cystic echinococcosis; AE, Alveolar echinococcosis; RR, Relative risk; LLR, Log-likelihood ratio.

## Discussion

The spatial and temporal epidemiological characteristics of the detection rates of newly diagnosed echinococcosis cases in the endemic areas of Sichuan Province from 2013 to 2024 were analyzed in this study. Spatial autocorrelation analysis was used to identify spatial clustering and the specific locations of these clusters, while spatiotemporal scan analysis was used to comprehensively analyze the spatiotemporal clusters of newly diagnosed echinococcosis cases from both temporal and spatial perspectives. These findings provide theoretical support for the prevention and control of echinococcosis in Sichuan Province.

The temporal distribution results revealed that the overall detection rate of newly diagnosed echinococcosis cases in Sichuan Province from 2013 to 2024 remained relatively high compared to the nationwide rate [16–19]. The annual detection rates of newly diagnosed echinococcosis cases, including cystic and alveolar types, demonstrated a declining trend every year, maintaining a low level since 2019. This trend is closely related to the comprehensive prevention and control strategy implemented in the endemic areas of Sichuan Province during this period, which focused on “controlling the source of infection, combined with health education, intermediate host management, and patient diagnosis and treatment” [20]. The decrease in the intensity of integrated prevention efforts could be due to a reduction in prevention and control funding from departments, including agriculture, water conservancy, and forestry, after the completion of the “13th Five-Year Plan” evaluation for echinococcosis control. Besides, collaboration among relevant departments has decreased, increasing the risk of an epidemic rebound. Moreover, the COVID-19 pandemic may have diverted the government’s attention from echinococcosis control, contributing to the rebound in detection rates. Heterogeneity in screening coverage between counties (e.g., 3-year vs. 5-year cycles) may partially influence detection rates, although our quality control framework minimizes diagnostic variability. From 2013 to 2021, the detection rate of CE cases was higher than that of AE cases; however, this trend reversed between 2022 and 2024. This epidemiological pattern likely stems from the distinctive transmission dynamics of CE, which predominantly follows a “livestock-dog-human living environment” transmission cycle within domestic environments. Through China’s central government transfer payment initiatives, we have established comprehensive intervention measures including household dog population control, regular anthelmintic treatment of canines, and proper sanitary disposal of canine feces. These coordinated interventions have successfully disrupted the parasite’s life cycle, leading to substantial reduction in CE transmission risks across endemic areas [21–23]. In contrast, AE transmission predominantly depends on a sylvatic cycle involving “wild canids-small mammals-human living environment” ecological interactions. While stray dog deworming programs have been implemented, comprehensive control measures remain unattainable for key wildlife reservoirs including foxes, wolves, and various small mammal intermediate hosts. Conservation policies prohibit population culling of these protected species, while logistical constraints prevent effective anthelmintic administration and subsequent fecal waste management in natural habitats. These ecological and regulatory barriers collectively hinder complete disruption of the AE transmission pathway [24–28]. After careful consideration of both scientific validity and operational practicality, we propose a two-pronged intervention strategy for AE control: First, implementing systematic and periodic collection of wild canid feces in human activity zones, with proper biosafety protocols for subsequent sterilization and disposal. This targeted environmental sanitation measure can significantly mitigate human exposure risks to AE pathogens. Second, we recommend implementing comprehensive health promotion campaigns in endemic regions focusing on strengthening community knowledge of AE prevention strategies through targeted education programs, and enhancing patient compliance with scheduled clinical monitoring through improved healthcare engagement mechanisms.

In terms of spatial distribution, areas with high detection rates of echinococcosis in Sichuan Province were mostly concentrated in the northwest, particularly in Baiyu, Shiqu, Dege, Aba, and Xinlong counties, while the southeastern regions exhibited lower detection rates. This finding is consistent with previous studies [29,30]. The high detection rates in the northwest may be associated with geographical, social, and ecological factors. Environmental factors, including high altitude, cold, low oxygen, and high ultraviolet conditions, slow the natural degradation of *Echinococcus* eggs while prolonging their survival in soil under fluctuating low temperatures and humidity. Experiments have demonstrated that eggs can survive for over a year at 0–10 °C [31–33]. The high grassland coverage (over 70%) and extensive grazing ranges in these areas facilitate the widespread dispersal of *Echinococcus eggs* through dog activities [34]. Social factors include the predominance of pastoralism, with an average of 3–5 dogs per household, low rates of dog feces disposal, and high dog infection rates. Yaks and Tibetan sheep are essential for pastoral livelihoods, and home slaughtering is common, with diseased organs often discarded or fed to dogs, perpetuating the parasite’s life cycle. Moreover, the lack of cold chain facilities, the prevalence of raw or air-dried meat consumption, low awareness of echinococcosis, and poor early healthcare-seeking behavior among pastoralists contribute to the high detection rates [35–40]. Ecological factors include the presence of Tibetan dogs, Tibetan foxes, wolves, yaks, and Tibetan sheep, all of which are hosts of *Echinococcus*. Infrared camera monitoring and fecal testing revealed a wildlife infection rate of approximately 12% in high-altitude areas, forming a “dog-wildlife-livestock” cross-transmission network. The high density of intermediate hosts, long distances between villages (more than 20 kilometers), and limited healthcare accessibility further exacerbate the high detection rates in these regions [41–43].

There are certain differences in the spatial distributions of CE and AE. While areas with high AE detection rates are also concentrated in the northwest, the specific counties differ from those of CE. For example, Rangtang County has emerged as one of the areas with the highest detection rates of AE, reflecting the distinct transmission cycles and spatial distribution patterns of the two types. This highlights the need for refined classification and targeted control strategies based on local epidemiological characteristics of the disease. The 47 mixed-infection cases clustered in seven northwestern counties suggest co-circulation of *E. granulosus* and *E. multilocularis* in overlapping zoonotic cycles. This may reflect behavioral factors (e.g., dogs preying on both livestock and rodents) or environmental contamination with both parasite eggs, warranting targeted surveys in these foci.

The detection rates of newly diagnosed echinococcosis cases have recently increased in Rangtang, Heishui, and Aba counties in the Aba Prefecture. This implies that although the overall situation of prevention and control is improving, there may still be gaps or new challenges in some areas that require further strengthening of the control measures.

The spatial clustering and temporal trends of newly diagnosed echinococcosis cases (overall, cystic, and alveolar) from 2013 to 2024 were analyzed using spatial autocorrelation analysis. Compared to the overall detection rates and those of AE, the spatial clustering of CE detection rates was more limited in terms of years, possibly due to its specific geographical distribution and host factors [44]. The spatial clustering of AE detection rates became more extensive over time, most likely because the average worm burden of *E. multilocularis* in dogs is higher than that of *E. granulosus*, and unlike that of *E. granulosus,* the infection rate of *E. multilocularis* does not decline with dog age. This may contribute to the broader spatial clustering of AE, as well as the stronger transmission capacity and diverse host range of *E. multilocularis* [45–47]. Local spatial autocorrelation analysis identified “high-high” clusters for overall, cystic, and AE detection rates, primarily concentrated in the northwest and north, including Shiqu, Dege, Ganzi, and Baiyu counties. These results are consistent with those of the spatial distribution. “Low-low” clusters were primarily found in the southeastern regions, where smaller-scale pastoralism and limited host diversity hinder the transmission cycle of echinococcosis [48]. Notably, “low-low” clusters were incompletely free of cases but had relatively lower detection rates than “high-high” clusters. The primary endemic foci of echinococcosis in Sichuan Province are concentrated in the northwestern region, particularly in Shiqu, Seda, Baiyu, and Dege counties Local governments have implemented comprehensive control measures including population-based screening and case treatment, management of intermediate and definitive hosts, and community health education programs. These interventions have led to significant reductions in both disease prevalence and transmission risks in the northwest. However, this study reveals substantial regional heterogeneity in detection rates across Sichuan. Spatial clustering analysis indicates an emerging epidemiological pattern, with northern regions demonstrating a distinct rebound trend. This is evidenced by the progressive formation of “high-high” clusters in northern Sichuan.

The local spatial autocorrelation results reveal that persistent “high-high” clusters in northwestern and northern Sichuan indicate these areas serve as epidemiological cores for echinococcosis transmission, closely associated with the local ecological transmission network of “dogs-wildlife-livestock”. For example, in counties like Shiqu and Dege, high infection rates in free-ranging pastoral dogs, combined with frequent cross-contact between wild animals (e.g., Tibetan foxes, wolves) and domestic yaks/sheep, form an unbroken transmission chain. The presence of “high-low” outliers, such as Ganzi County surrounded by high-risk areas, suggests that localized control measures (e.g., canine deworming, health education) have shown initial efficacy. However, the risk of pathogen spillover from adjacent high-risk zones remains a concern. “Low-high” outliers reflect a complex scenario: southeastern low-prevalence areas like Tianquan County bordering high-risk counties may experience pathogen introduction due to livestock trade or seasonal pastoral migration. These regions often have blind spots in prevention and control, such as insufficient ultrasound diagnostic capacity in grassroots healthcare facilities or inadequate implementation of dog management policies, leading to delayed detection of imported cases. Additionally, while “low-low” clusters exhibit overall low risk, sporadic cases may exist in remote villages with poor transportation, indicating potential underreporting in these areas.

Considering the transmission characteristics of echinococcosis, the spatial heterogeneity highlighted above underscores the need for refined prevention strategies. For “high-high” clusters, strengthen surveillance of wildlife hosts and standardized dog management, and pilot closed-loop measures like “dog registration-regular deworming-fecal harmless treatment”. For “high-low” and “low-high” zones, enhance cross-regional joint prevention and control by adding quarantine stations at livestock markets and implementing mobile screenings for migrant herders. This spatial pattern-based intervention approach can more precisely allocate prevention resources, improving disease control efficiency under the Healthy China 2030 initiative.

Spatiotemporal scan analysis of the overall, CE, and AE detection rates from 2013 to 2024 revealed the clustering characteristics of echinococcosis across different types, times, and locations. All primary clusters for overall, CE, and AE detection rates were located in the northwest, including Shiqu, Seda, Baiyu, Ganzi, and Dege counties. This implies that the northwestern region remains a high-risk area for echinococcosis, requiring special attention. The clustering centers for different echinococcosis detection rates were geographically close, demonstrating the stability of echinococcosis distribution in space [16]. The primary clustering period for the overall and CE detection rates was 2013–2016, while for AE, it extended to 2018. This suggests that the transmission and prevalence of echinococcosis during this period may have been influenced by different factors, including climate, host distribution, and human activities. Secondary clusters were identified in Heishui county (for overall detection rates), Ruoergai county (for CE detection rates), and Kangding county (for overall and AE detection rates).

The high-risk zones for echinococcosis in Sichuan Province are predominantly distributed along its border regions with Qinghai Province and Tibet autonomous region, specifically within the high-altitude ecosystems of the Qinghai-Tibet Plateau. This spatial distribution pattern shows significant consistency with epidemiological findings from neighboring Qinghai Province and Tibet autonomous region, collectively forming China’s most critical endemic foci for echinococcosis. Our analysis identifies several shared cross-regional risk determinants: traditional livestock husbandry practices, limited healthcare infrastructure, persistent wildlife reservoir populations, and distinctive plateau geographical characteristics [49,50]. In addition, our study revealed a distinct temporal pattern of echinococcosis in Sichuan Province that diverged from both national and global trends. Between 2013 and 2024, while the overall detection rate of echinococcosis in Sichuan demonstrated a consistent decline, a notable epidemiological shift occurred in 2022 when the detection rate of AE surpassed that of CE. This inversion of the typical AE-CE ratio contrasts sharply with patterns observed in other mixed-endemic regions worldwide, where CE cases consistently outnumber AE reports.

The epidemiological characteristics of echinococcosis in Sichuan Province were elucidated in this study. However, it has some limitations. The data may deviate from the actual incidence due to underreporting or misdiagnosis caused by insufficient technical capacity at the grassroots level and imperfect reporting systems. The lack of data on the natural, biological, and socioeconomic factors influencing echinococcosis transmission limits the comprehensive analysis of spatiotemporal clustering. Future studies should incorporate local natural conditions, socioeconomic factors, cultural backgrounds, and resident habits for a comprehensive understanding.

Based on these findings, we recommend implementing targeted prevention and control strategies in areas with different risk levels. For “high-high” clusters, efforts should be directed toward controlling transmission hosts, such as implementing dog registration and standardized management, reducing the number of stray dogs, ensuring proper disposal of dog feces, promoting centralized livestock slaughter, enforcing inspection and quarantine measures, and regulating the movement of animals and animal products. In areas endemic for AE, rodent control should be tailored to local conditions to reduce transmission risks. Enhanced surveillance, interdepartmental collaboration, and public participation are essential to sustain the “government-led, department-coordinated, and public-involved” prevention mechanism. Health education should target key populations, including monks, teachers, students, and slaughterhouse workers, integrating echinococcosis prevention knowledge into community, school, and temple education programs. Large-scale population screening may no longer be cost-effective in “low-low” clusters, and efforts should shift to disease monitoring and health education, focusing on high-risk townships to prevent resurgence or spread of the disease.

## Conclusions

Through a 12-year continuous monitoring and multi-scale spatial analysis of echinococcosis in Sichuan Province, this study demonstrated that the disease is persistently highly prevalent in the northwest, with increased risks of AE and emergence of secondary epidemic foci. These findings provide a basis for formulating targeted prevention and control strategies. We recommend adopting differentiated strategies for “high-high” and “low-low” clusters, establishing a “spatial warning-targeted intervention-dynamic evaluation” closed-loop management system, incorporating spatiotemporal scan results into annual prevention plans, strengthening surveillance, interdepartmental collaboration, and health education, promoting interdisciplinary research, and developing AI-based ultrasound diagnostic systems to improve data quality and contribute to the goals of Healthy China 2030.

## Author covntributions

**Conceptualization:** Wei He, Liying Wang, Qian Wang.

**Data curation:** Wei He, Wenjie Yu, Sha Liao, Qi Wang, Ruirui Li.

**Formal analysis:** Wei He, Wenjie Yu, Sha Liao, Guangjia Zhang.

**Funding acquisition:** Yan Huang, Qian Wang.

**Investigation:** Wei He, Wenjie Yu, Guangjia Zhang, Qi Wang, Renxin Yao.

**Methodology:** Wei He, Liying Wang, Qian Wang.

**Project administration:** Wei He, Yang Liu, Bo Zhong, Qian Wang.

**Resources:** Wei He, Liying Wang, Qian Wang.

**Software:** Wei He, Sha Liao, Zhongshuang Zhang.

**Supervision:** Yan Huang, Liying Wang, Qian Wang.

**Validation:** Wei He, Yang Liu, Bo Zhong, Yan Huang.

**Visualization:** Wei He, Qi Wang, Ruirui Li, Liu Yang.

**Writing – original draft:** Wei He, Liying Wang, Qian Wang.

**Writing – review & editing:** Wei He, Yang Liu, Bo Zhong, Liying Wang, Qian Wang.
